# *Lacrimispora sanguinis* sp. nov., isolated from human blood

**DOI:** 10.1371/journal.pone.0334875

**Published:** 2025-10-31

**Authors:** Hui-Jin Yu, Yun Young Cho, Jayoung Paek, Minhee Kang, Mi Young Ahn, Heejung Kim, Jung-Hyun Byun, Tae Yeul Kim, Hee Jae Huh, Lu Bai, Young-Hyo Chang

**Affiliations:** 1 Department of Laboratory Medicine, Seoul Medical Center, Seoul, Republic of Korea; 2 Department of Laboratory Medicine and Genetics, Samsung Medical Center, Sungkyunkwan University School of Medicine, Seoul, Republic of Korea; 3 Access and Benefit-Sharing Research Support Center, Korea Research Institute of Bioscience and Biotechnology, Daejeon, Republic of Korea; 4 Biomedical Engineering Research Center, Smart Healthcare Research Institute, Samsung Medical Center, Seoul, Republic of Korea; 5 Department of Internal Medicine, Seoul Medical Center, Seoul, Republic of Korea; 6 Department of Laboratory Medicine, Gyeongsang National University Hospital, Gyeongsang National University College of Medicine, Jinju, Republic of Korea; 7 Department of Medical Device Management and Research, Samsung Advanced Institute for Health Sciences & Technology, Sungkyunkwan University, Seoul, Republic of Korea; Universidad Autonoma de Chihuahua, MEXICO

## Abstract

A rod-shaped, obligate anaerobic, Gram-stain-positive bacterium isolated from the human blood was designated as the strain HJ-01^T^. Analysis of the 16S rRNA gene sequence revealed that the strain HJ-01^T^ belonged to the genus *Lacrimispora*, and was most closely related to *L. celerecrescens* strains DSM 105336 and MCM B-936, with both 99.3% similarity. The average nucleotide identity values between the strain and the most closely related type strains ranged from 75.3% to 91.4%, while the values between the strain and the two non-type strains of *L. celerecrescens*, DSM 105336 and MCM B-936, were 98.8% to 98.9%. The digital DNA-DNA hybridization values between the strain and the most closely related type strains ranged from 19.8% to 44.5%, whereas the values between the strain and *L. celerecrescens* strains DSM 105336 and MCM B-936 were 89.7% to 91.6%. The phylogenomic analysis revealed that the strain formed a cluster adjacent to *L. celerecrescens* strains DSM 105336 and MCM B-936. The main fatty acids identified were C_16:0_ and C_18:1_ *cis* 11 DMA. The cell wall contained the *meso*-diaminopimelic acid-based peptidoglycan. The end products of the fermentation were acetic acid and formic acid. The strain HJ-01^T^ and the related *Lacrimispora* strains shared similar antibiotic resistance profiles, including high resistance to clindamycin (8–256 µg ml−1), linked to the *cfr*(C) gene located within a 3,378-bp chromosomal transposed unit. Given the chemotaxonomic, phenotypic, and phylogenetic properties, HJ-01^T^ (= KCTC 25933^T^ = JCM 37550^T^) represent a novel species of the genus *Lacrimispora*, for which the name *Lacrimispora sanguinis* sp. nov. is proposed. Additionally, we suggest that *L. celerecrescens* DSM 105336 and MCM B-936 be transferred to *Lacrimispora sanguinis* sp. nov.

## Introduction

The *Clostridium sphenoides* group has recently been reclassified as the genus *Lacrimispora* within the family *Lachnospiraceae* [1]. This genus currently comprises 11 species with validly published names [2], with *Lacrimispora sphenoides* serving as the type species, and the most recent addition being *Lacrimispora sinapis*, isolated from pickled potherb mustard [3]. Members of the genus *Lacrimispora* are generally Gram-positive, spore-forming, rod-shaped, and anaerobic bacteria [1]. They are commonly isolated from environmental sources such as soil, plants, animal, and human sources, with some occasionally implicated in human infections. For instance, *L. sphenoides* has been reported to cause gastroenteritis, osteomyelitis, peritonitis, and bloodstream infections [4–8], while *Lacrimispora celerecrescens* has been associated with osteomyelitis [9,10].

Microorganisms play vital roles in all ecosystems, yet their rapid and accurate characterization remains a major challenge. The matrix-assisted laser desorption/ionization time-of-flight mass spectrometry (MALDI-TOF MS) has become a powerful microbial classification technique due to its speed, affordability, and ease of use, making it exceptionally valuable for detecting bacteria in clinical, environmental, and microbiome diversity research [11]. Previously, we isolated a strain HJ-01^T^ from human blood and identified it within the *Lacrimispora* genus by MALDI-TOF MS. The aim of the present study was to determine the taxonomic status of this strain using a polyphasic approach.

## Materials and methods

### Ethics statement

This study was reviewed and approved by the Institutional Review Board of Seoul Medical Center (approval no. SEOUL 2024-08-007). The requirement for informed consent was waived as the study involved only bacterial strains obtained through routine diagnostic testing and public culture collections, with all patient data fully anonymized. Patient data were retrospectively accessed for research purposes from 01/10/2024 to 30/11/2024.

### Strain isolation and patient history

Strain HJ-01^T^ was isolated from the blood of an 81-year-old woman with hypertension and no other significant comorbidities. Following intravenous infusion of an albumin solution not authorized for clinical use by the Korean Ministry of Food and Drug Safety at the time, she was presented to the emergency room with signs of sepsis. Her initial vital signs were: body temperature of 39.6°C, blood pressure of 92/47 mm Hg, pulse rate of 108 beats per minute, respiratory rate of 22 breaths per minute, and oxygen saturation of 92% while breathing ambient air. Two sets of blood cultures were obtained, and empirical antibiotic therapy with meropenem was initiated. After overnight incubation, Gram-positive bacilli were detected in the anaerobic blood culture bottles, prompting the addition of teicoplanin. The anerobic, Gram-positive bacilli were subcultured on Brucella blood agar supplemented with 5% (v/v) sheep blood and incubated anaerobically at 36°C for 3 days. Strain HJ-01^T^ was identified using MALDI-TOF MS (MALDI Biotyper; Bruker, Bremen, Germany) and preserved in skim milk (BD Difco Skim Milk) at −80°C until further use. Upon availability of the identification result, teicoplanin was discontinued, while meropenem was continued, resulting in a favorable clinical response. Subsequent blood cultures were negative, and the patient recovered fully, being discharged on day 15 without complications.

### Phylogenetic analysis

16S rRNA gene amplification was performed using primers previously reported by Bai et al. [12]. The 16S rRNA gene sequence of the strain was aligned with reference sequences of published prokaryotic type strains retrieved from the GenBank and EzBioCloud databases (https://www.ezbiocloud.net/) [13]. The phylogenetic trees were generated from unambiguous alignments, with alignment conducted with PHYLIP and phylogenetic analyses carried out using jPHYDIT [14]. Whole-genome and core gene phylogenies were constructed using FastME (GBDP, distance formula d5) and UBCG2, respectively, with 100 and 1,000 bootstrap replicates. *Lactonifactor longoviformis* DSM 17459^T^ was designated as the outgroup, and branch lengths were scaled to 0.20 substitutions per site [15,16]. Maximum-likelihood (ML), neighbor-joining (NJ), and minimum-evolution (ME) algorithms were used to reconstruct phylogenetic trees [17]. Reliability of the phylogenetic tree was assessed by bootstrap analysis with 1000 resamplings [18]. *Lactonifactor longoviformis* DSM 17459^T^ was used as the outgroup.

### Genomic analysis

Genomic DNA of strain HJ-01^T^ was extracted using the Wizard HMW DNA Extraction Kit following the manufacturer’s protocol. Library preparation utilized the SMRTbell protocol, with size selection (7–12 kb cutoff) performed using the Megaruptor 3 (Diagenode). Sequencing was conducted on the PacBio Revio platform (Pacific Biosciences) by Phyzen Genomics Institute (Seongnam, Republic of Korea). De novo assembly of high-fidelity (HiFi) reads was performed with Flye (v2.9.4), and assembly validation was achieved by aligning HiFi reads to assembled sequences using Pbmm2 (v1.14.0). Genome annotation was carried out using Prokka (v1.14.6). The genome map visualization and genome comparison were generated with the proksee server [19]. CheckM was used to evaluate the completeness of the genome [20]. Average nucleotide identity (ANI) values were determined using OrthoANIu [21]. Digital DNA-DNA hybridization (dDDH) values were calculated using the formula 2 of the Genome-to-Genome Distance Calculator (GGDC, version 3.0; http://ggdc.dsmz.de/ggdc.php#) [22]. In addition, a multi-locus species tree based on 100 conserved core genes, derived from the whole-genome sequences of the strain and its closely related strains, was generated using the IQ-TREE maximum likelihood method implemented in autoMLST (https://automlst.ziemertlab.com) [23]. Comprehensive Antibiotic Resistance Database (CARD) was utilized to detect antibiotic resistance genes [24]. PathogenFinder (v1.1) and VirulenceFinder (v3.0.2) were used to identify pathogenicity and virulence genes [25]. Whole-genome sequences and their corresponding GenBank accession numbers, obtained from NCBI (https://www.ncbi.nlm.nih.gov/genome), were used for ANI, dDDH, and phylogenomic analyses.

### Phenotypic and chemotaxonomic analyses

The strain and its related strains were cultivated on Brucella blood plates at 36°C for 3 days prior to biochemical, physiological, and morphological characterization. For aerotolerance test, the strain was further incubated in fluid thioglycollate medium at 36°C for 3 days [26]. Cells motility was examined in semi-solid Brucella serum medium (0.3% w/v). Flagellation and cell morphology were observed using a transmission electron microscope (CM20, Philips, Netherlands) and a phase-contrast microscope (E600, Nikon, Japan). Gram-staining was carried out using a commercial Gram-staining kit (BioMérieux, France) according to the manufacturer’s instructions. Following cultivation on Brucella blood plates at 36°C for 3 days, spore formation was assessed by staining with 5% malachite green and counterstaining with safranin [27]. Gram-staining and spore formation were observed using an upright microscope (ECLIPSE Ci, Nikon, Japan). The Brucella blood medium was employed to assess tolerance to pH, temperature, and salinity. pH tolerance was tested over a range of 5.0 to 12.0 in 1-unit increments, salinity tolerance was evaluated using NaCl concentration of up to 5% (w/v), in 0.5% increments and growth temperature was determined from 4 to 60°C in 5–10°C increments [28]. Additional biochemical phenotypic characterization was preformed using API 20A and 32A kits (BioMérieux, France).

Fermentation end products were analyzed by liquid chromatography (Ultimate 3000, Thermo Dionex, USA) using an Aminex 87H column (300 × 10 mm, Bio-Rad, USA) with0.01N H_2_SO_4_ as the mobile phase at flow rate of 0.5 ml/min. Detection was performed at 210 nm using a RefractoMAX520 detector (Japan) [29]. Whole-cell fatty acid composition was analyzed after culturing the strain and reference strains on Brucella blood plates at 36°C for 3 days, followed by cell harvesting. Fatty acids profiles were determined by gas chromatography using the MOORE6 Library (version 6.0) of the MIDI/Hewlett-Packard Microbial Identification System [30]. Peptidoglycan composition was examined according to the method outlined by Schumann [31].

Antimicrobial susceptibility testing was conducted using the Etest (bioMérieux, Marcy-l’Étoile, France) following the manufacturer’s instructions against penicillin, ampicillin, amoxicillin-clavulanate, piperacillin-tazobactam, clindamycin, ertapenem, imipenem, and metronidazole. Minimum inhibitory concentrations (MICs) were interpreted according to the breakpoints outlined in the Clinical and Laboratory Standards Institute M100-Ed34 document [32].

## Results and discussion

### Phylogenetic analysis

**Fig 1:**
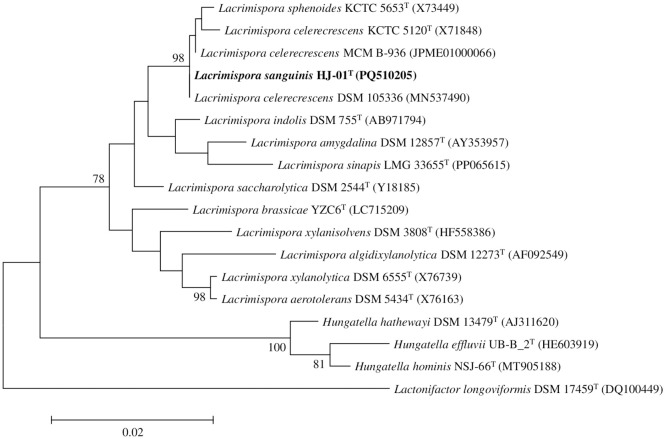
Phylogenetic consensus tree based on 16S rRNA gene sequences, reconstructed with the maximum-likelihood (ML) method, indicating the taxonomic positions of strain HJ-01^T^ and its close relatives. Bootstrap values (≥70%) based on 1,000 replicates are shown at branch nodes. *Lactonifactor longoviformis* DSM 17459^T^ was used as an outgroup. Bar, 0.02 substitutions per nucleotide.

The 1,461 bp 16S rRNA gene sequence of the strain HJ-01^T^ was used for phylogenetic analysis. The 16S rRNA similarities between the strain and the most closely related strains, *L. celerecrescens* DSM 105336 and *L. celerecrescens* MCM B-936, were both 99.3% (S1 Table). The ML tree placed the strain within a cluster of the genus *Lacrimispora* (Fig 1) forming a distinct branch adjacent to *L. celerecrescens* and *L. sphenoides*. Phylogenetic analysis with the use of the ML method demonstrated that the strain belongs to the genus *Lacrimispora*. Similar results were observed with the NJ (S1 Fig) and ME trees (S2 Fig).

### Genomic analysis

Overall, 921,496,507 bp of the strain HJ-01^T^ (170 × coverage) was read. The genome assembly of the strain HJ-01^T^ consisted of a single contig of 5,411,172 bp, with an N50 of 5,411,172 bp. The sequenced genome satisfied the minimal standards for quality [34]. The genome completeness of the strain was 98.1%, with 2.3% contamination. The total genome length of the strain HJ-01^T^ was 5,411,172 bp, with a G + C content of 43.2 mol% (Table 1). The G + C content of the strain was comparable to that of other species within *Lacrimispora*. The assembled genome of strain HJ-01^T^ contained 5,055 genes, including 4,928 protein-coding sequences (CDS), 18 rRNAs, and 69 tRNAs (S5 Fig).

**Table 1:** Genomic characteristics of strain HJ-01^T^and closely related species.

Attribute	Accession no.	Coverages (×)	N50 (bp)	Mean size (bp)	Genome size (bp)	G + C content (mol%)	Contigs	Genes	CDSs	rRNAs	tRNAs
**1**	CP173277	170	5,411,172	5,411,172	5,411,172	43.2	1	5,055	4,928	18	69
**2**	VUMC00000000	300	713,749	217850.1	5,446,253	43.0	25	5,008	4,927	10	67
**3**	JPME00000000	83	208,566	54760.99	5,038,011	43.5	92	4,286	4,036	27	72
**4**	LT630003	159	5,300,235	5,300,235	5,300,235	43.8	1	4,869	4,662	18	68
**5**	PGET00000000	85	5,272,838	5,272,838	5,272,838	43.9	1	4,841	4,625	21	72
**6**	BRYH00000000	191	216,902	93,224	6,059,566	44.6	65	5,832	5,526	1	64
**7**	JHWJ00000000	−	213,027	676053	4,732,373	42.4	7	4,397	4,276	8	65
**8**	AZUI00000000	−	6,383,701	6,383,701	6,383,701	44.9	1	5,837	5,701	18	71
**9**	MCIA00000000	10	233,695	132,569	4,639,933	40.2	35	4,225	4,081	2	63
**10**	BRPJ00000000	217	108,220	51,944	5,350,244	42.3	103	5,097	5,038	2	58
**11**	CP002109	30	4,662,871	4,662,871	4,662,871	45.0	1	4,389	4,154	18	68
**12**	PTJA00000000	174	318,361	170,583	5,629,247	41.9	33	5,353	5,277	6	62
**13**	BRPI00000000	237	484,068	220,038	4,620,800	41.9	21	4,366	4,318	1	15
**14**	CP141832	100	3,753,647	3,753,647	3,753,647	44.0	1	3,525	3,390	18	66

Strains: 1, HJ-01^T^, 2, *L. celerecrescens* DSM 105336, 3, *L. celerecrescens* MCM B-936, 4, *L. sphenoides* KCTC 5653^T^, 5, *L. celerecrescens* KCTC 5120^T^, 6, *L. brassicae* YZC6^T^, 7, *L. aerotolerans* DSM 5434^T^, 8, *L. indolis* DSM 755^T^, 9, *L. algidixylanolytica* DSM 12273^T^, 10, *L. amygdalina* DSM 12857^T^, 11, *L. saccharolytica* DSM 2544^T^, 12, *L. xylanisolvens* DSM 3808^T^, 13, *L. xylanolytica* DSM 6555^T^, and 14, *L. sinapis* LMG 33655^T^. − , not detected.

**Fig 2:**
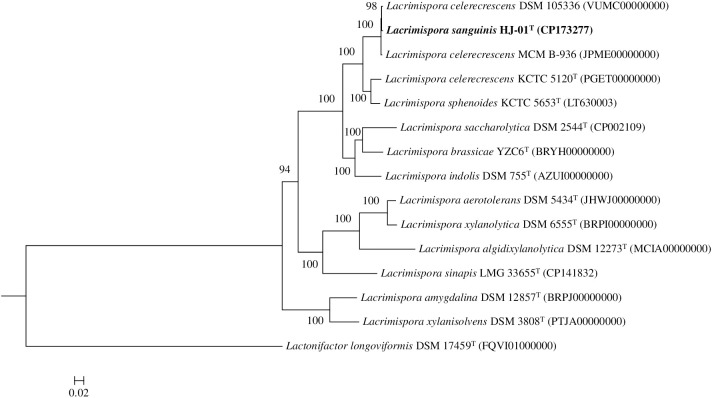
Molecular phylogenetic analysis based on genome sequences of strain HJ-01^T^ and its close relatives using autoMLST with default parameters (>100 core genes). Bootstrap values (≥70%) based on 1,000 replicates are shown at branch nodes. *Lactonifactor longoviformis* DSM 17459^T^ was served as an outgroup. Bar, 0.02 substitutions per nucleotide position.

The genome comparison demonstrated that the strain was most closely related to *L. celerecrescens* DSM 105336 and *L. celerecrescens* MCM B-936 (S3-S6 Fig). The phylogenomic tree revealed that the strain HJ-01^T^ formed a distinct clade with *L. celerecrescens* strains DSM 105336 and MCM B-936. This clade was positioned adjacent to *L. celerecrescens* KCTC 5120^T^ and *L. sphenoides* KCTC 5653^T^ (Fig 2). The ANI values between the strain HJ-01^T^ and the most closely related type strains ranged from 75.3% to 91.4%, while the values between the strain and the two non-type strains of *L. celerecrescens*, DSM 105336 and MCM B-936, were 98.9% and 98.8%, respectively. The dDDH values between the strain and the most closely related type strains ranged from 19.8% to 44.5%, whereas the values between the strain and *L. celerecrescens* strains DSM 105336 and MCM B-936 were 91.6% and 89.7%, respectively (Table 2). The ANI and dDDH values indicated that the values between the strain and the related type strains within the genus *Lacrimispora* were below the bacterial species delineation thresholds [33]. Moreover, the strain HJ-01^T^ was most closely related to the *L. celerecrescens* strains DSM 105336 and MCM B-936. These results indicate that the strain represents a new species within the genus *Lacrimispora*, with the likelihood that *L. celerecrescens* DSM 105336 and *L. celerecrescens* MCM B-936 also belongs to this novel species.

**Table 2:** Average nucleotide identity (ANI) and digital DNA-DNA hybridization (dDDH) values (%) between strain HJ-01^T^ and the close relatives of *Lacrimispora.*

No.	1	2	3	4	5	6	7	8	9	10	11	12	13	14
1	**100**	**91.6**	**89.7**	44.5	43.9	30.2	28.4	20.2	19.8	20.9	29.7	20.1	20.4	21.5
2	**98.9**	**100**	**89.6**	44.4	43.6	30.5	29.7	28.0	20.9	20.2	20.2	20.0	20.2	19.6
3	**98.8**	**98.8**	**100**	44.8	44.0	30.4	29.8	28.0	21.1	20.2	20.2	20.1	20.2	19.8
4	91.4	91.4	91.4	**100**	56.0	31.4	30.5	28.6	21.6	20.5	20.2	20.4	20.3	19.9
5	91.1	91.0	91.2	94.1	**100**	31.4	30.7	28.7	21.6	20.3	20.2	20.7	20.5	20.0
6	85.0	85.3	85.1	85.7	85.9	**100**	35.9	35.7	21.4	21.5	20.5	20.5	20.5	20.0
7	84.9	84.9	84.9	85.4	85.4	75.8	**100**	31.8	21.6	20.8	20.1	20.7	20.3	20.0
8	84.2	84.0	84.0	84.4	84.5	87.9	86.2	**100**	21.9	21.4	19.9	20.7	20.1	20.2
9	77.4	77.1	77.2	77.5	77.4	77.2	77.2	77.5	**100**	19.9	21.9	20.0	21.8	21.3
10	76.3	76.3	76.0	76.1	76.1	75.3	76.1	76.3	75.8	**100**	19.4	32.5	19.3	19.1
11	75.9	76.1	76.0	85.7	75.9	75.8	75.7	75.8	79.2	75.2	**100**	20.2	57.6	25.8
12	75.9	76.0	75.9	75.9	75.9	75.9	75.8	75.9	75.6	87.0	75.6	**100**	19.5	19.5
13	75.8	75.8	75.9	75.8	75.9	75.9	75.7	75.5	79.0	75.4	94.5	75.2	**100**	25.5
14	75.3	75.2	75.3	85.4	75.2	75.3	74.9	75.0	78.0	74.7	82.8	74.9	82.6	**100**

Strains: 1, HJ-01^T^, 2, *L. celerecrescens* DSM 105336, 3, *L. celerecrescens* MCM B-936, 4, *L. sphenoides* KCTC 5653^T^, 5, *L. celerecrescens* KCTC 5120^T^, 6, *L. brassicae* YZC6^T^, 7, *L. indolis* DSM 755^T^, 8, *L. saccharolytica* DSM 2544^T^, 9, *L. sinapis* LMG 33655^T^, 10, *L. amygdalina* DSM 12857^T^, 11, *L. aerotolerans* DSM 5434^T^, 12, *L. xylanisolvens* DSM 3808^T^, 13, *L. xylanolytica* DSM 6555^T^, and 14, *L. algidixylanolytica* DSM 12273^T^. The values on the upper right are the dDDH values and the values on the lower left are the ANI values. Bold character, values higher than the bacterial species delineation thresholds.

CARD analysis identified the *cfr*(C) gene in the genomes of the strain HJ-01^T^ and related strains, strongly associated with high clindamycin resistance. The *cfr* gene encodes a methyltransferase that modifies the C8 position of A2503 in 23S rRNA, conferring resistance to phenicols, lincosamides, oxazolidinones, pleuromutilins, and streptogramin A (PhLOPSA phenotype). Since its initial discovery in *Staphylococcus sciuri* in 2000, five *cfr* variants (*cfr*, *cfr*(B), *cfr*(C), *cfr*(D), and *cfr*(E)) have been described [34]. The *cfr*(C) gene has been reported in *Campylobacter coli*, *Clostridioides difficile*, *Clostridium perfringens*, and *Bacteroides fragilis*, typically located on plasmids or chromosomes associated with transposable elements [34–37]. The identification of *cfr*(C) in the *Lacrimispora* genus is novel, representing the first report of this gene in the genus. This gene was located on the chromosome within a 3,378-bp transposed unit, flanked by two mismatched direct repeats (S7 Fig), suggesting at least two independent transposition events. The absence of transposase-encoding genes within 10 kb upstream or downstream of the transposed unit indicates its stabilization as a chromosomal feature.

Genome analysis using PathogenFinder and VirulenceFinder predicted strain HJ-01^T^ to be a non-human pathogen (0.227 probability) and no pathogenicity or virulence genes were detected. Interestingly, HJ-01^T^ matched 17 non-pathogenic protein families, all of which were also found in *L. celerecrescens* DSM 105336. These included 5 conserved hypothetical protein, 1 type II secretion system, 1 nucleotideyl transferase, 1 transcriptional regulator, 1 CdaR, 1 binding-protein-dependent transport systems inner membrane component, 1 ribosomal protein L17, 1 stage III sporulation protein AD, 1 protein of unknown function (DUF77), 1 ribosomal protein S6, 1 DNA-directed RNA polymerase omega subunit, 1 phosphotransferase system, 1 phosphocarrier protein HPr, and 1 protein of unknown function (DUF1540). The clinical outcomes further supported the predicted non-pathogenicity of the strain, as the patient recovered fully following appropriate antibiotic treatments. Although strain HJ-01^T^ appears to be non-pathogenic, its isolation from a clinical sample suggests clinical relevance, particularly in the context of polymicrobial communities or opportunistic colonization in compromised hosts. Similar observations have been reported for *L. celerecrescens*, which has been identified as an opportunistic pathogen in humans, primarily in post-traumatic or wound-related infections with low known pathogenicity and no identified virulence genes [38,39].

### Phenotypic and chemotaxonomic analyses

The closely related strains *L. celerecrescens* DSM 105336, *L*. *celerecrescens* KCTC 5120^T^, and *L*. *sphenoides* KCTC 5653^T^ were used as reference strains. Strain HJ-01^T^ exhibited characteristic features of the genus *Lacrimispora* [1], being rod-shaped, spore-forming, motile, and Gram-stain-positive (S8 Fig). The spore was terminal in position and oval in shape (S9 Fig). Aerotolerance testing confirmed that the strain HJ-01^T^ is an obligate anaerobe. The strain grew at 15–37°C (optimal at 35–37°C), pH range of 6–9 (optimal at 7) and withstood salinity levels of up to 1.5% (w/v). API 20A revealed that HJ-01^T^ was ferments lactose, and raffinose, which was different from *L. sphenoides* KCTC 5653^T^. API 32A revealed that HJ-01^T^ and *L. celerecrescens* DSM 105336 were negative to α-galactosidase activity, unlike *L. celerecrescens* KCTC 5120^T^ and *L. sphenoides* KCTC 5653^T^. The strain hydrolyzed esculin but did not produce indole and urease. Detailed phenotypic characteristics of the strain and related strains are presented in Table 3.

**Table 3:** Distinguishing phenotypic characteristics between strain HJ-01T and closely related species.

Characteristic	1	2	3	4
Isolated from:	Human blood	Wild boar	Wound infections	Methanogenic culture started with a cow manure inoculum
Growth at:				
pH 9	+	−	+	+
pH 10	−	−	+	−
pH 11	−	−	+	−
Acid production from:				
Lactose	+	−	−	−
Raffinose	+	+	−	+
α-Galactosidase	−	−	+	+
Acid production (mg/L):				
Acetic acid	892.7	826.5	958.7	790.6
Formic acid	230.6	305.7	178.5	202.4
G + C content (mol%)	43.2	43.0	43.8	43.9

Strains: 1, HJ-01^T^, 2, *L. celerecrescens* DSM 105336; 3, *L. sphenoides* KCTC 5653^T^; and 4, *L. celerecrescens* KCTC 5120^T^. + , Positive; − , negative.

The strain HJ-01^T^ produced acetic acid (892.7 mg/L), and formic acid (230.6 mg/L) as the major metabolic end products, similar to the related strains. The predominant whole-cell fatty acids (mean value > 10% of total fatty acids) were C_16:0_ (26.1%), and C_18:1_ *cis* 11 DMA (11.0%). These were also major components in *L. celerecrescens* DSM 105336 and *L. sphenoides* KCTC 5653^T^, albeit at different percentages, whereas C_18:1_ *cis* 11 DMA was absent among the dominant whole-cell fatty acid in *L. celerecrescens* KCTC 5120^T^ (Table 4). Analysis of cell wall peptidoglycan indicated that the strain and its closely related strains contained *meso*-diaminopimelic acid (*meso*-Dpm) as the diagnostic amino acid. The MIC results for strain HJ-01^T^ and the reference strains showed susceptibility to most of the tested antibiotics, including penicillin, ampicillin, amoxicillin-clavulanate, piperacillin-tazobactam, ertapenem, imipenem, and metronidazole (S2 Table), aligning with previous studies [7,8,10]. However, all the strains were resistant to clindamycin. Collectively, the phenotypic and chemotaxonomic characteristics distinguished the strain from its closely related strains.

**Table 4:** Cellular fatty acid profiles of strain HJ-01T and closely related species.

Fatty acid	1	2	3	4
C_14:0_	4.6	4.6	3.0	5.0
C_14:0_ DMA	tr	1.0	tr	1.3
C_15:0_	2.4	2.1	tr	2.3
C_16:0_	26.1	26.9	32.4	26.8
C_16:0_ ALDE	tr	1.2	1.3	1.5
C_16:0_ DMA	4.3	5.0	4.5	6.1
C_16:1_ *cis* 7	2.3	2.4	1.3	2.3
C_16:1_ *cis* 9	7.8	8.2	4.9	8.0
C_16:1_ *cis* 9 DMA	6.3	5.7	3.5	6.9
C_17:0_	1.2	1.2	−	1.3
C_18:0_ DMA	1.2	1.3	1.7	1.5
C_18:0_	1.6	1.7	2.7	2.8
C_18:1_ *cis* 9	4.2	4.3	5.3	3.7
C_18:1_ *cis* 9 DMA	7.0	7.1	9.8	8.4
C_18:1_ *cis* 11 DMA	11.0	10.3	11.7	7.8
Summed Feature 4*	2.3	2.3	1.7	2.9
Summed Feature 6*	1.2	tr	tr	1.1
Summed Feature 7*	1.7	1.7	3.0	2.1
Summed Feature 8*	3.3	3.2	3.9	2.3
Summed Feature 10*	6.6	6.2	5.7	3.3

Strains: 1, HJ-01^T^; 2, *L. celerecrescens* DSM 105336; 3, *L. sphenoides* KCTC 5653^T^; and 4, *L. celerecrescens* KCTC 5120^T^. Values are percentage of total fatty acid detected. tr, trace amount (<1.0%); − , not detected.

*Summed Features are fatty acids that cannot be resolved reliably from another fatty acid using the chosen chromatographic conditions. The MIDI system groups these fatty acids together as one feature with a single percentage of the total. Summed Feature 4: unknown 14.762 and/or C_15:2_ and/or C_15:1_ *cis* 7; Summed Feature 6: C_15:0_ anteiso 3OH and/or C_16:1_ *cis* 7 DMA; Summed Feature 7: C_17:2_ at 16.760 and/or C_17:1_ *cis* 8; Summed Feature 8: C_17:1_ *cis* 9 and/or C_17:2_ at 16.801; Summed Feature 10: C_18:1_ c11/t9/t6 and/or unknown 17.834.

In conclusion, the representative peptidoglycan type of the strain HJ-01^T^ was *meso*-Dpm, consistent with that of related strains within the genus *Lacrimispora*. The end products of the fermentation were acetic acid and formic acid, and the main cellular fatty acids were C_16:0_ and C_18:1_ *cis* 11 DMA, which was similar to the related strains within the genus *Lacrimispora*. 16S rRNA phylogenetic analysis showed that the strain formed a distinct cluster within the *Lacrimispora* genus. The G + C content of the HJ-01^T^ genome was 43.2 mol%. The ANI values between the strain and the related species in *Lacrimispora* ranged from 75.3% to 91.4%, while the values between the strain and *L. celerecrescens* strains DSM 105336 and MCM B-936 were 98.8–98.9%. The dDDH values between the strain and the related species in *Lacrimispora* ranged from 19.8% to 44.5%, whereas the values between the strain and *L. celerecrescens* strains DSM 105336 and MCM B-936 were 89.7–91.6%. Therefore, the strain HJ-01^T^ represents a new species within the genus *Lacrimispora*, and the name *Lacrimispora sanguinis* sp. nov. is proposed. Additionally, our data suggest that *L. celerecrescens* DSM 105336 and MCM B-936 may also belong to *Lacrimispora sanguinis* sp. nov.

The strain was isolated from human blood, and while intravenous infusion may represent a possible route of entry, the infection pathway remains uncertain. As an opportunistic microorganism, host factors such as patient age may have contributed to infection; however, it cannot be confirmed that the bacterium directly caused the observed clinical symptoms.

This study underscores the significance of identifying *L. sanguinis* sp. nov., contributing to a broader understanding of the microbial diversity associated with a hospitalized patient. Furthermore, it highlights the value of genome-based taxonomy in delineating species boundaries and guiding future research into the ecological roles and potential clinical significance of novel anaerobic bacteria.

### Description of *Lacrimispora sanguinis* sp. nov.

*Lacrimispora sanguinis* (san’gui.nis. L. n. *sanguis*, blood; referring to the strain isolation from human blood).

Cells are obligately anaerobic, Gram-stain-positive, spore-forming, rod-shaped, and motile by means of peritrichous flagella. Cells measure 0.5–1.0 µm in diameter, and 1.3–4.1 μm long. Colonies grown anaerobically on Brucella blood agar (5% sheep blood) at 36°C for 3 days are cream-colored. Cells grow in the range 15–37°C, at pH 6–9, with optimal growth at 35−37°C and pH 7. The species tolerance of NaCl is up to 1.5% (w/v). The end products are acetic acid and formic acid. Based on the API 20A, the strain ferments glucose, mannitol, lactose, maltose, salicin, xylose, arabinose, celiobiose, mannose, raffinose, rhamnose, and trehalose; but does not ferment saccharose, gelatine, glycerol, melezitose, and sorbitol. Based on the API 32A, the strain produces β-galactosidase, α-glucosidase, β-glucosidase, β-arabinosidase, glutamic acid, and alkaline phosphatase; but do not produce arginine dihydrolase, α-galactosidase, β-galactosidase-6-phosphate, β-glucuronidase, *N*-acetyl-β-glucosaminidase, D-raffinose, D-mannose, α-fucosidase, reduction of nitrates, arginine arylamidase, proline arylamidase, phenylalanine arylamidase, leucyl-glycine arylamidase, leucine arylamidase, glycine arylamidase, pyroglutamic acid arylamidase, alanine arylamidase, tyrosine arylamidase, histidine arylamidase, serine arylamidase, and glutamyl -glutamic acid arylamidase activities. Esculin is hydrolyzed, whereas indole and urease are not produced. The strain is catalase- and oxidase-negative. The predominant fatty acids are C_16:0_ and C_18:1_ *cis* 11 DMA. The cell-wall peptidoglycan contains *meso*-diaminopimelic acid as a diagnostic amino acid. The strain is susceptible to penicillin, ampicillin, amoxicillin-clavulanate, piperacillin-tazobactam, ertapenem, imipenem, and metronidazole, but resistant to clindamycin.

Strain HJ-01^T^ (KCTC 25933^T^ = JCM 37550^T^) was isolated from human blood. Its genome is 5,411,172 bp in length, with a G + C content of 43.2 mol%.

## Supporting information

S1 Fig Phylogenetic consensus tree based on 16S rRNA gene sequence of strain HJ-01^T^, reconstructed with the neighbor-joining (NJ), indicating the taxonomic positions of isolate and close relatives. Bootstrap values (≥70%) based on 1,000 subsets are shown at branch nodes. *Lactonifactor longoviformis* DSM 17459^T^ was used as an outgroup. Bar, 0.02 substitutions per nucleotide.
(DOCX)

S2 Fig Phylogenetic consensus tree based on 16S rRNA gene sequence of strain HJ-01^T^, reconstructed with the minimum-evolution (ME), indicating the taxonomic positions of isolate and the close relatives. Bootstrap values (≥70%) based on 1,000 subsets are shown at branch nodes. *Lactonifactor longoviformis* DSM 17459^T^ was used as an outgroup. Bar, 0.02 substitutions per nucleotide.
(DOCX)

S3 Fig Phylogenomic tree based on whole genome sequences showing the relationships between strain HJ-01^T^ and its closely related strains within the genus *Lacrimispora.* Tree inferred with FastME 2.1.6.1 [40] from GBDP distances calculated from genome sequences. The branch lengths are scaled in terms of GBDP distance formula *d*_5_. The numbers above branches are GBDP pseudo-bootstrap support values > 60% from 100 replications, with an average branch support of 98.4%. The tree was rooted at the midpoint [41].
(DOCX)

S4 Fig Phylogenomic tree based on core gene sequence by UBCG2 showing the relationships between strain HJ-01^T^ and its closely related strains within the genus *Lacrimispora.* Bootstrap values based on 1000 replications are listed as percentages at branch points. Bar, 0.20 substitutions per site. *Lactonifactor longoviformis* DSM 17459^T^ was used as an outgroup.
(DOCX)

S5 Fig A circular genome map of strain HJ-01ᵀ. The second and third inner circles display the G + C content and G + C skew, respectively.
(DOCX)

S6 Fig Genome comparison of strain HJ-01ᵀ and closely related strains within the genus *Lacrimispora.* Starting from the innermost ring, rings 1 and 2 represent the GC skew (purple/green) and GC content (black) of strain HJ-01ᵀ. Rings 3 and 4 display protein-coding genes (purple), tRNA genes (orange), tmRNA genes (green), rRNA genes (pink), and repeat regions (blue) on the forward and reverse strands of strain HJ-01ᵀ. The remaining rings show genome comparisons of *L. celerecrescens* DSM 105336 (ring 5), *L. celerecrescens* MCM B-936 (ring 6), *L. sphenoides* KCTC 5653ᵀ (ring 7), and *L. celerecrescens* KCTC 5120^T^ (ring 8) with strain HJ-01ᵀ.
(DOCX)

S7 Fig Schematic presentation of the vicinity of the *cfr*(C) gene in the chromosome of strain HJ-01ᵀ. Solid arrows indicate the positions and orientations of the open reading frames, and their colors are based on the estimated function of encoded proteins. Arrow heads indicate direct repeats.
(DOCX)

S8 Fig Scanning electron micrograph (SEM) and transmission electron micrograph (TEM) of the strain HJ-01^T^. A, SEM image of HJ-01^T^ (bar, 5 µm); and B, TEM image of HJ-01^T^ (bar, 1 µm).
(DOCX)

S9 Fig Transmission electron micrograph (TEM) of the strain HJ-01^T^ endospore formation and spore. A, TEM image of HJ-01^T^ endospore (bar, 500 nm); and B, TEM image of HJ-01^T^ spore (bar, 200 nm).
(DOCX)

S1 Table 16S rRNA similarities between strain HJ-01^T^ and the close relatives of *Lacrimispora.* Strains: 1, HJ-01^T^; 2, *L. celerecrescens* DSM 105336; 3, *L. celerecrescens* MCM B-936; 4, *L. celerecrescens* KCTC 5120^T^; 5, *L. sphenoides* KCTC 5653^T^; 6, *L. indolis* DSM 755^T^; 7, *L. saccharolytica* DSM 2544^T^; 8, *L. brassicae* YZC6^T^; 9, *L. amygdalina* DSM 12857^T^; 10, *L. xylanolytica* DSM 6555^T^; 11, *L. aerotolerans* DSM 5434^T^; 12, *L. sinapis* LMG 33655^T^; 13, *L. xylanisolvens* DSM 3808^T^; 14, *L. algidixylanolytica* DSM 12273^T^.
(DOCX)

S2 Table Antimicrobial susceptibility patterns of strain HJ-01^T^ and its closely related strains. Strains: 1, *L*. *sanguinis* HJ-01^T^; 2, *L. celerecrescens* DSM 105336; 3, *L. sphenoides* KCTC 5653^T^; 4, *L. celerecrescens* KCTC 5120^T^. All data were obtained from the current study. S, susceptible; I, intermediate; R, resistant.
(DOCX)
