# Supplementary material for: Lacrimispora sanguinis sp. nov., isolated from human blood
Source: PLoS One. 2025 Oct 31;20(10):e0334875. doi: 10.1371/journal.pone.0334875 (PMC12578346; doi:10.1371/journal.pone.0334875)

**S2 Fig. Phylogenetic consensus tree based on 16S rRNA gene sequence of strain HJ-01^T^, reconstructed with the minimum-evolution (ME), indicating the taxonomic positions of isolate and the close relatives.** Bootstrap values (≥70%) based on 1,000 subsets are shown at branch nodes. *Lactonifactor longoviformis* DSM 17459^T^ was used as an outgroup. Bar, 0.02 substitutions per nucleotide.


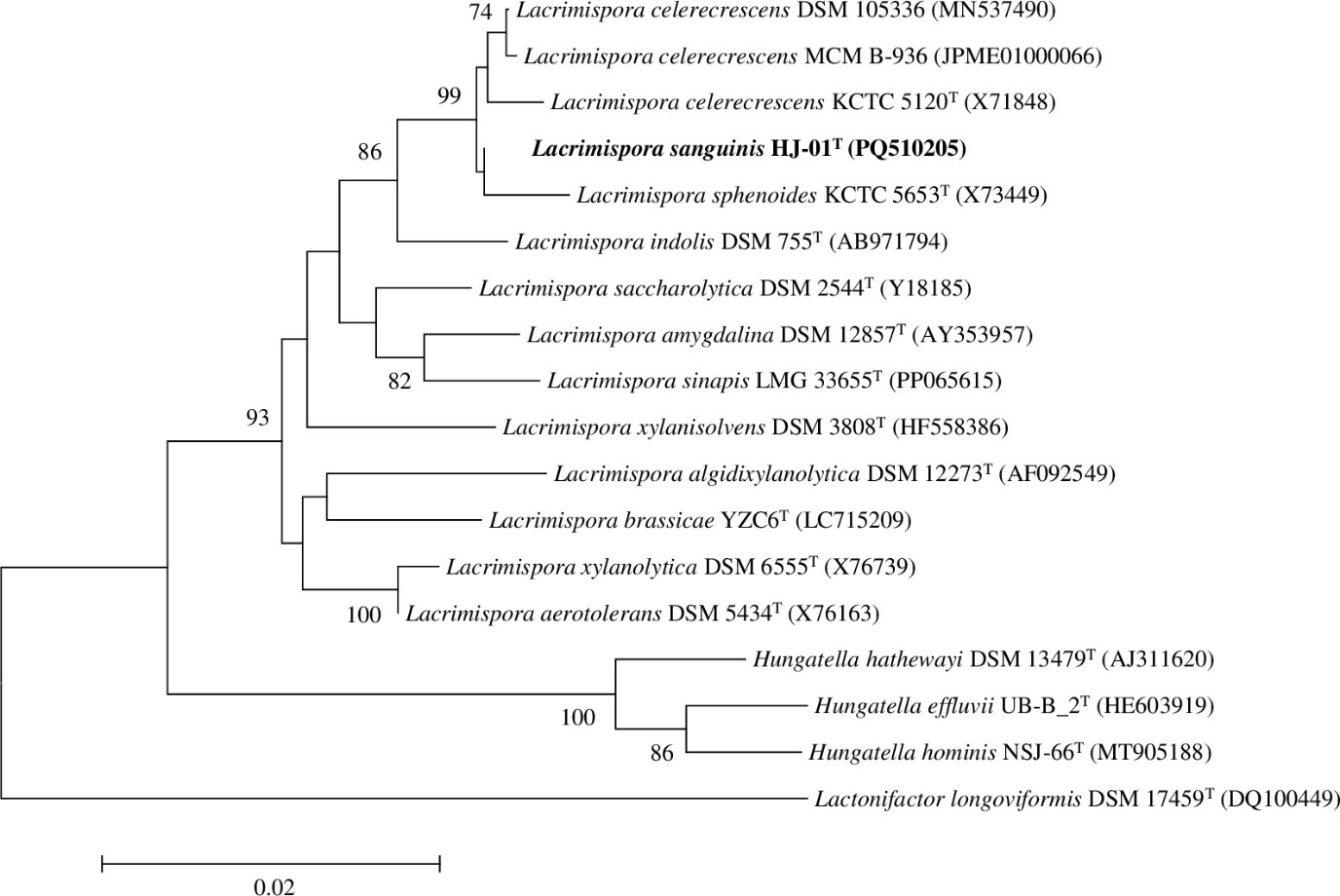

Supplement: S2 Fig — Bootstrap values (≥70%) based on 1,000 subsets are shown at branch nodes. Lactonifactor longoviformis DSM 17459T was used as an outgroup. Bar, 0.02 substitutions per nucleotide. (DOCX) [file pone.0334875.s002.docx]
