# Supplementary material for: Lacrimispora sanguinis sp. nov., isolated from human blood
Source: PLoS One. 2025 Oct 31;20(10):e0334875. doi: 10.1371/journal.pone.0334875 (PMC12578346; doi:10.1371/journal.pone.0334875)
Supplement: S3 Fig — Tree inferred with FastME 2.1.6.1 [40] from GBDP distances calculated from genome sequences. The branch lengths are scaled in terms of GBDP distance formula d5. The numbers above branches are GBDP pseudo-bootstrap support values > 60% from 100 replications, with an average branch support of 98.4%. The tree was rooted at the midpoint [41]. (DOCX) [file pone.0334875.s003.docx]

**S3 Fig. Phylogenomic tree based on whole genome sequences showing the relationships between strain HJ-01^T^ and its closely related strains within the genus *Lacrimispora.*** Tree inferred with FastME 2.1.6.1 [40] from GBDP distances calculated from genome sequences. The branch lengths are scaled in terms of GBDP distance formula *d*_5_. The numbers above branches are GBDP pseudo-bootstrap support values > 60 % from 100 replications, with an average branch support of 98.4 %. The tree was rooted at the midpoint [41].

**
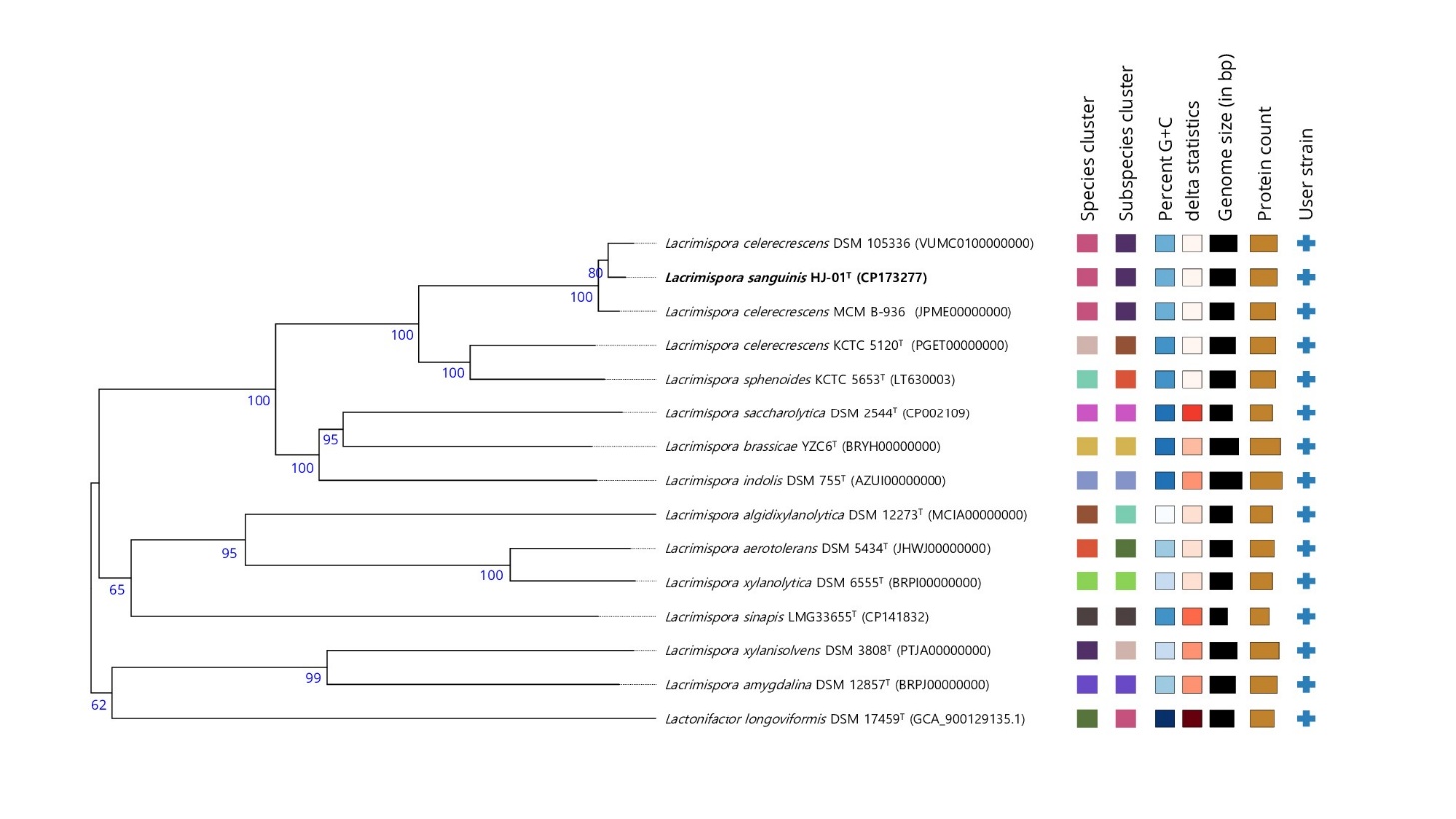
**
