# Supplementary material for: Lacrimispora sanguinis sp. nov., isolated from human blood
Source: PLoS One. 2025 Oct 31;20(10):e0334875. doi: 10.1371/journal.pone.0334875 (PMC12578346; doi:10.1371/journal.pone.0334875)
Supplement: S4 Fig — Bootstrap values based on 1000 replications are listed as percentages at branch points. Bar, 0.20 substitutions per site. Lactonifactor longoviformis DSM 17459T was used as an outgroup. (DOCX) [file pone.0334875.s004.docx]

**S4 Fig. Phylogenomic tree based on core gene sequence by UBCG2 showing the relationships between strain HJ-01^T^ and its closely related strains within the genus *Lacrimispora*.** Bootstrap values based on 1000 replications are listed as percentages at branch points. Bar, 0.20 substitutions per site. *Lactonifactor longoviformis* DSM 17459^T^ was used as an outgroup.

**
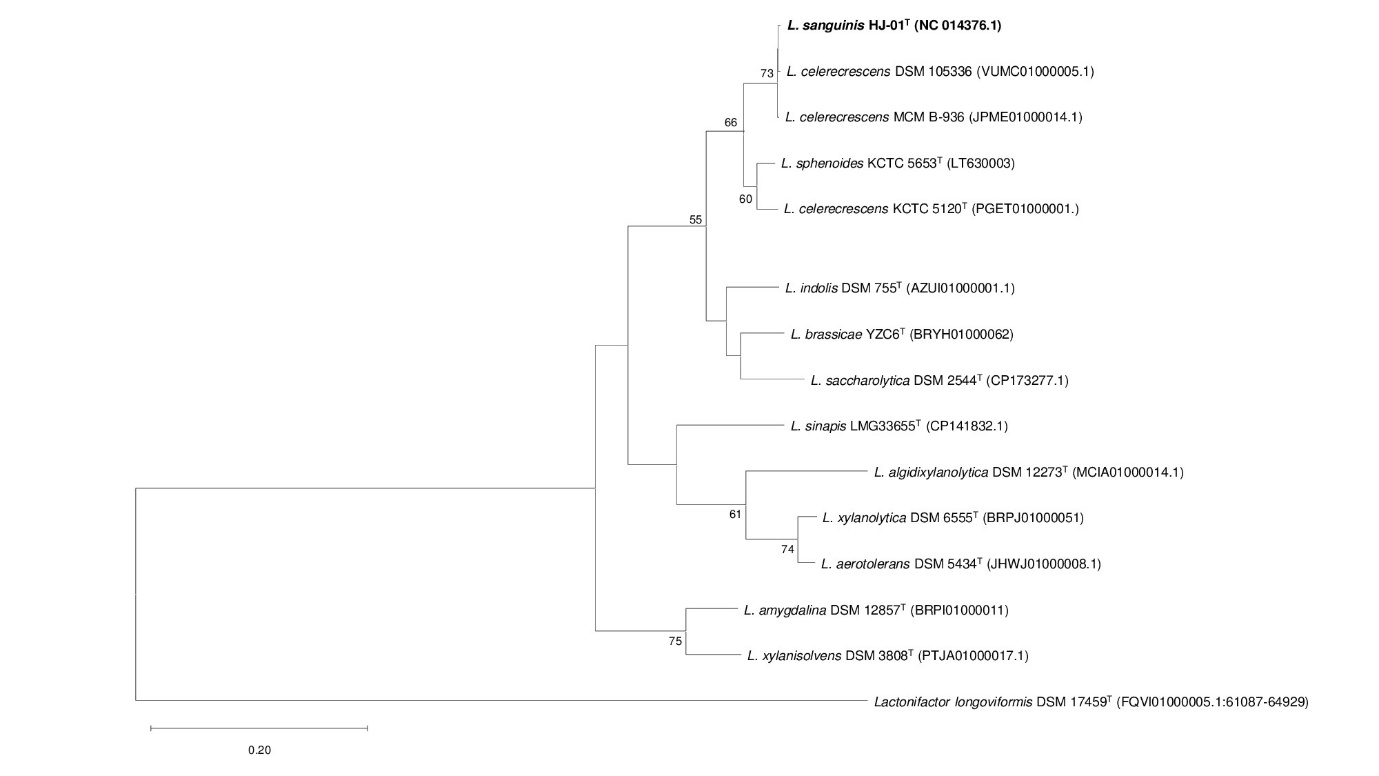
**
