# Supplementary material for: Lacrimispora sanguinis sp. nov., isolated from human blood
Source: PLoS One. 2025 Oct 31;20(10):e0334875. doi: 10.1371/journal.pone.0334875 (PMC12578346; doi:10.1371/journal.pone.0334875)

**S5 Fig.** **A circular genome map of strain HJ-01ᵀ. The outermost circle represents coding sequences (CDSs), followed by tRNA genes, tmRNA genes, and rRNA genes in sequential order.** The second and third inner circles display the G+C content and G+C skew, respectively.


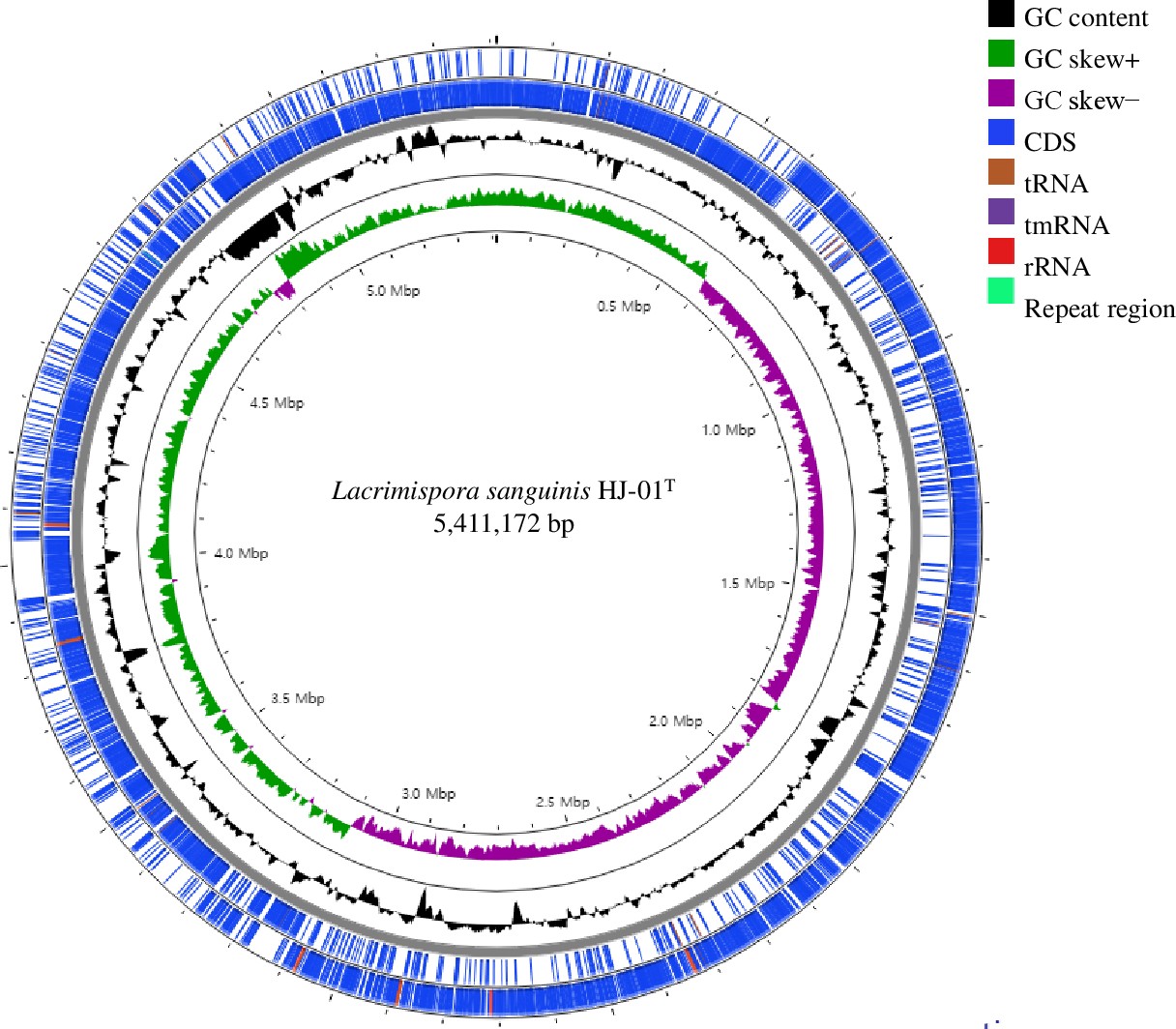

Supplement: S5 Fig — The second and third inner circles display the G + C content and G + C skew, respectively. (DOCX) [file pone.0334875.s005.docx]
