# Supplementary material for: Lacrimispora sanguinis sp. nov., isolated from human blood
Source: PLoS One. 2025 Oct 31;20(10):e0334875. doi: 10.1371/journal.pone.0334875 (PMC12578346; doi:10.1371/journal.pone.0334875)

**S6 Fig.** **Genome comparison of strain HJ-01ᵀ and closely related strains within the genus *Lacrimispora*.** Starting from the innermost ring, rings 1 and 2 represent the GC skew (purple/green) and GC content (black) of strain HJ-01ᵀ. Rings 3 and 4 display protein-coding genes (purple), tRNA genes (orange), tmRNA genes (green), rRNA genes (pink), and repeat regions (blue) on the forward and reverse strands of strain HJ-01ᵀ. The remaining rings show genome comparisons of *L. celerecrescens* DSM 105336 (ring 5), *L. celerecrescens* MCM B-936 (ring 6), *L. sphenoides* KCTC 5653ᵀ (ring 7), and *L. celerecrescens* KCTC 5120^T^ (ring 8) with strain HJ-01ᵀ.


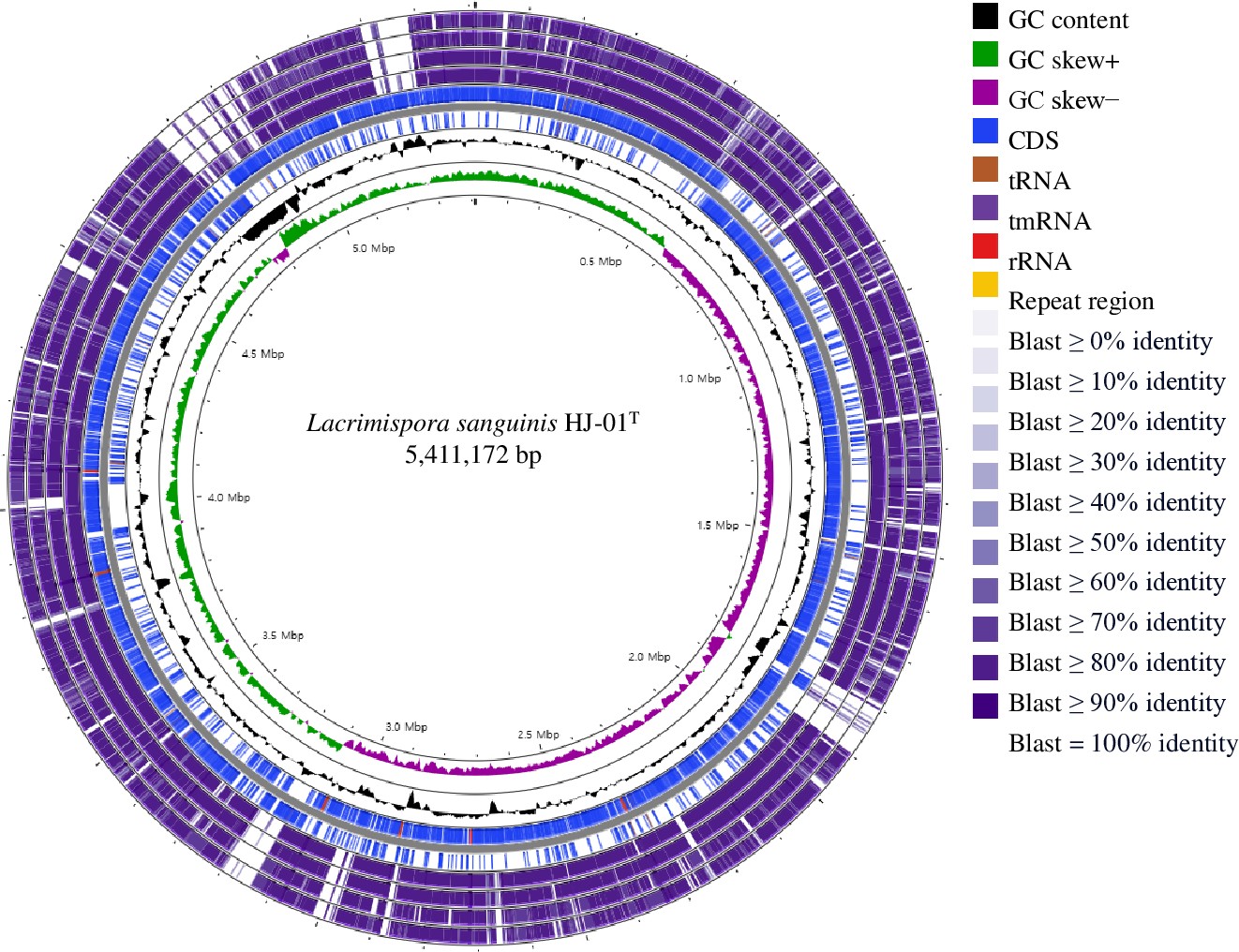

Supplement: S6 Fig — Starting from the innermost ring, rings 1 and 2 represent the GC skew (purple/green) and GC content (black) of strain HJ-01ᵀ. Rings 3 and 4 display protein-coding genes (purple), tRNA genes (orange), tmRNA genes (green), rRNA genes (pink), and repeat regions (blue) on the forward and reverse strands of strain HJ-01ᵀ. The remaining rings show genome comparisons of L. celerecrescens DSM 105336 (ring 5), L. celerecrescens MCM B-936 (ring 6), L. sphenoides KCTC 5653ᵀ (ring 7), and L. celerecrescens KCTC 5120T (ring 8) with strain HJ-01ᵀ. (DOCX) [file pone.0334875.s006.docx]
