# Supplementary material for: Lacrimispora sanguinis sp. nov., isolated from human blood
Source: PLoS One. 2025 Oct 31;20(10):e0334875. doi: 10.1371/journal.pone.0334875 (PMC12578346; doi:10.1371/journal.pone.0334875)

**S7 Fig.** **Schematic presentation of the vicinity of the *cfr*(C) gene in the chromosome of strain HJ-01ᵀ.** Solid arrows indicate the positions and orientations of the open reading frames, and their colors are based on the estimated function of encoded proteins. Arrow heads indicate direct repeats.


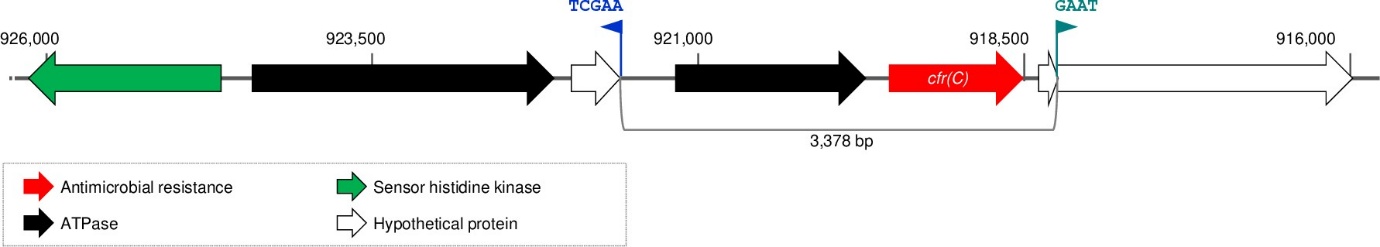

Supplement: S7 Fig — Solid arrows indicate the positions and orientations of the open reading frames, and their colors are based on the estimated function of encoded proteins. Arrow heads indicate direct repeats. (DOCX) [file pone.0334875.s007.docx]
