# Supplementary material for: Lacrimispora sanguinis sp. nov., isolated from human blood
Source: PLoS One. 2025 Oct 31;20(10):e0334875. doi: 10.1371/journal.pone.0334875 (PMC12578346; doi:10.1371/journal.pone.0334875)

**S8 Fig.** **Scanning electron micrograph (SEM) and transmission electron micrograph (TEM) of the strain HJ-01^T^.** A, SEM image of HJ-01^T^ (bar, 5 µm); and B, TEM image of HJ-01^T^ (bar, 1 µm).


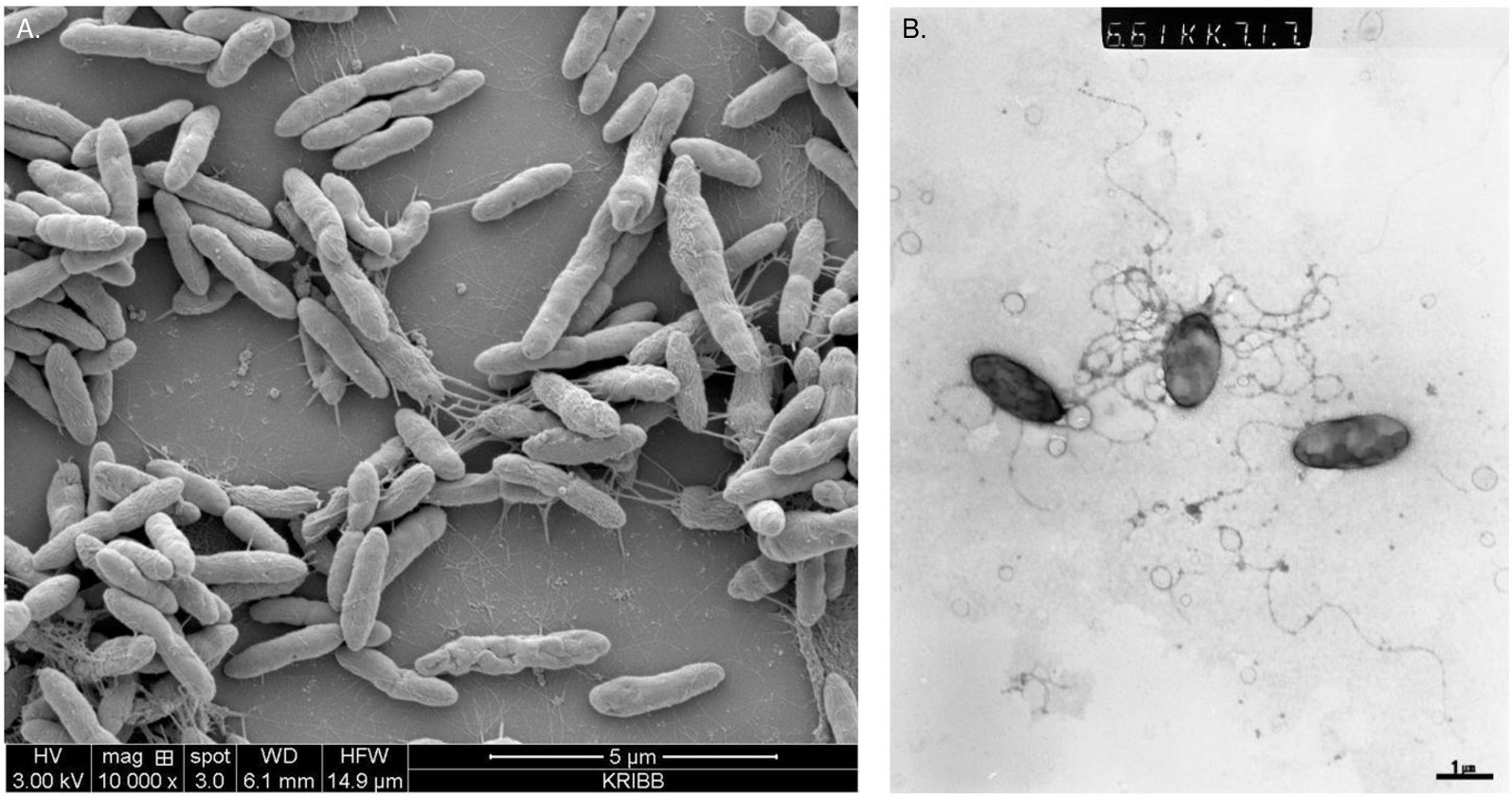

Supplement: S8 Fig — A, SEM image of HJ-01T (bar, 5 µm); and B, TEM image of HJ-01T (bar, 1 µm). (DOCX) [file pone.0334875.s008.docx]
