# Supplementary material for: Lacrimispora sanguinis sp. nov., isolated from human blood
Source: PLoS One. 2025 Oct 31;20(10):e0334875. doi: 10.1371/journal.pone.0334875 (PMC12578346; doi:10.1371/journal.pone.0334875)

**S9 Fig.** T**ransmission electron micrograph (TEM) of the strain HJ-01^T^ endospore formation and spore.** A, TEM image of HJ-01^T^ endospore (bar, 500 nm); and B, TEM image of HJ-01^T^ spore (bar, 200 nm).


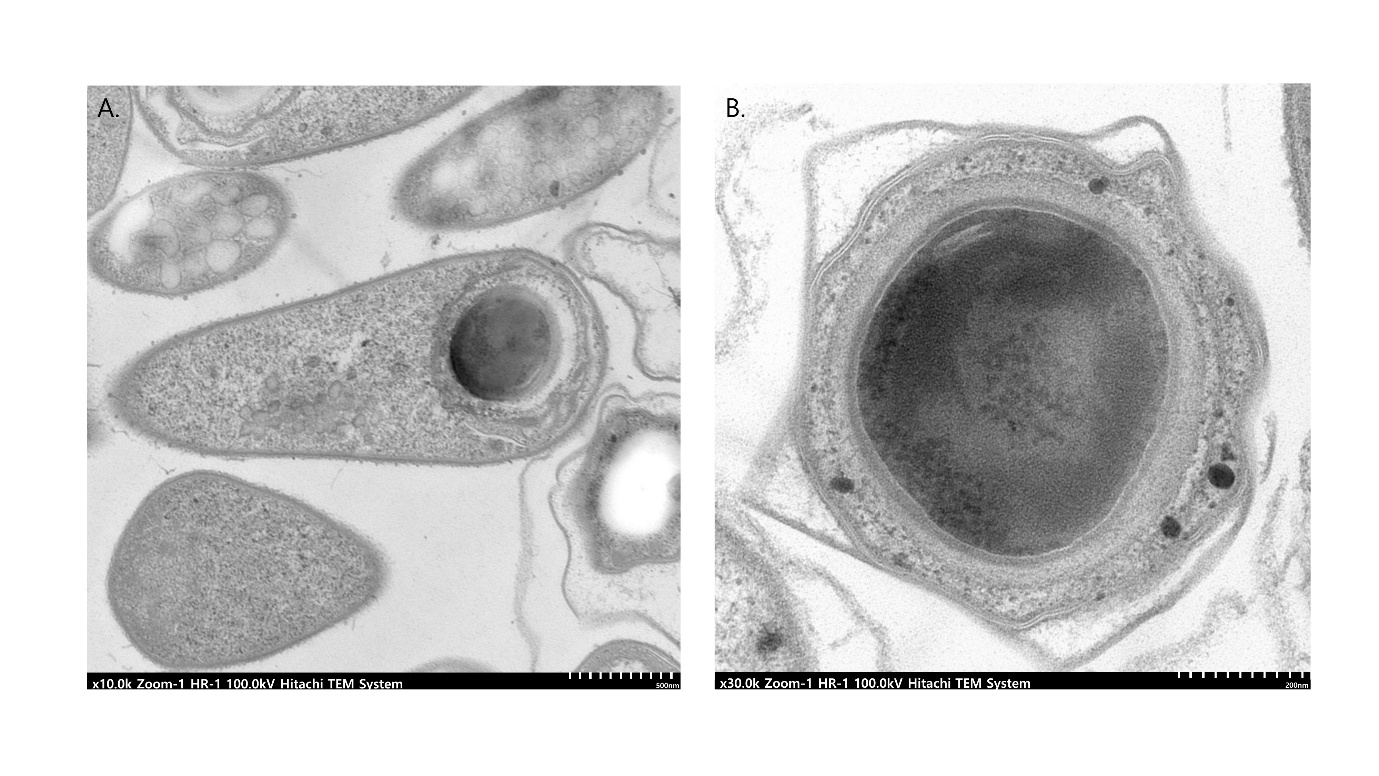

Supplement: S9 Fig — A, TEM image of HJ-01T endospore (bar, 500 nm); and B, TEM image of HJ-01T spore (bar, 200 nm). (DOCX) [file pone.0334875.s009.docx]
