# Supplementary material for: Lacrimispora sanguinis sp. nov., isolated from human blood
Source: PLoS One. 2025 Oct 31;20(10):e0334875. doi: 10.1371/journal.pone.0334875 (PMC12578346; doi:10.1371/journal.pone.0334875)
Supplement: S1 Table — Strains: 1, HJ-01T; 2, L. celerecrescens DSM 105336; 3, L. celerecrescens MCM B-936; 4, L. celerecrescens KCTC 5120T; 5, L. sphenoides KCTC 5653T; 6, L. indolis DSM 755T; 7, L. saccharolytica DSM 2544T; 8, L. brassicae YZC6T; 9, L. amygdalina DSM 12857T; 10, L. xylanolytica DSM 6555T; 11, L. aerotolerans DSM 5434T; 12, L. sinapis LMG 33655T; 13, L. xylanisolvens DSM 3808T; 14, L. algidixylanolytica DSM 12273T. (DOCX) [file pone.0334875.s010.docx]

**S1 Table.** **16S rRNA similarities between strain HJ-01^T^ and the close relatives of *Lacrimispora*.**

Strains: 1, HJ-01^T^; 2, *L. celerecrescens* DSM 105336; 3, *L. celerecrescens* MCM B-936; 4, *L. celerecrescens* KCTC 5120^T^; 5, *L. sphenoides* KCTC 5653^T^; 6, *L. indolis* DSM 755^T^; 7, *L. saccharolytica* DSM 2544^T^; 8, *L. brassicae* YZC6^T^; 9, *L. amygdalina* DSM 12857^T^; 10, *L. xylanolytica* DSM 6555^T^; 11, *L. aerotolerans* DSM 5434^T^; 12, *L. sinapis* LMG 33655^T^; 13, *L. xylanisolvens* DSM 3808^T^; 14, *L. algidixylanolytica* DSM 12273^T^.

| No. | 1 | 2 | 3 | 4 | 5 | 6 | 7 | 8 | 9 | 10 | 11 | 12 | 13 | 14 |
| --- | --- | --- | --- | --- | --- | --- | --- | --- | --- | --- | --- | --- | --- | --- |
| 1 | 100 |  |  |  |  |  |  |  |  |  |  |  |  |  |
| 2 | 99.3 | 100 |  |  |  |  |  |  |  |  |  |  |  |  |
| 3 | 99.3 | 99.9 | 100 |  |  |  |  |  |  |  |  |  |  |  |
| 4 | 98.7 | 99.3 | 99.3 | 100 |  |  |  |  |  |  |  |  |  |  |
| 5 | 98.2 | 98.7 | 98.4 | 98.1 | 100 |  |  |  |  |  |  |  |  |  |
| 6 | 98.2 | 98.6 | 98.5 | 98.3 | 97.7 | 100 |  |  |  |  |  |  |  |  |
| 7 | 98.1 | 98.5 | 98.5 | 98.3 | 97.3 | 98.4 | 100 |  |  |  |  |  |  |  |
| 8 | 97.7 | 98.0 | 97.9 | 97.5 | 96.9 | 97.8 | 98.1 | 100 |  |  |  |  |  |  |
| 9 | 97.5 | 97.8 | 97.9 | 97.7 | 96.8 | 98.4 | 98.9 | 97.5 | 100 |  |  |  |  |  |
| 10 | 97.3 | 97.7 | 97.6 | 97.2 | 97.0 | 97.5 | 97.9 | 97.9 | 97.5 | 100 |  |  |  |  |
| 11 | 97.3 | 97.5 | 97.6 | 97.3 | 96.6 | 97.4 | 98.0 | 97.8 | 97.6 | 99.0 | 100 |  |  |  |
| 12 | 97.1 | 97.5 | 97.4 | 97.4 | 96.7 | 97.9 | 98.3 | 97.2 | 98.7 | 97.7 | 97.7 | 100 |  |  |
| 13 | 97.1 | 97.4 | 97.4 | 97.3 | 96.4 | 97.8 | 98.1 | 97.6 | 98.0 | 97.8 | 98.0 | 97.9 | 100 |  |
| 14 | 96.9 | 97.2 | 97.1 | 97.0 | 96.3 | 96.7 | 97.7 | 97.8 | 97.5 | 97.8 | 97.8 | 97.9 | 97.8 | 100 |
