# Supplementary material for: Lacrimispora sanguinis sp. nov., isolated from human blood
Source: PLoS One. 2025 Oct 31;20(10):e0334875. doi: 10.1371/journal.pone.0334875 (PMC12578346; doi:10.1371/journal.pone.0334875)
Supplement: S2 Table — Strains: 1, L. sanguinis HJ-01T; 2, L. celerecrescens DSM 105336; 3, L. sphenoides KCTC 5653T; 4, L. celerecrescens KCTC 5120T. All data were obtained from the current study. S, susceptible; I, intermediate; R, resistant. (DOCX) [file pone.0334875.s011.docx]

**S2 Table. Antimicrobial susceptibility patterns of strain HJ-01^T^ and its closely related strains*.***

Strains: 1, *L*. *sanguinis* HJ-01^T^; 2, *L. celerecrescens* DSM 105336; 3, *L. sphenoides* KCTC 5653^T^; 4, *L.* *celerecrescens*KCTC 5120^T^. All data were obtained from the current study. S, susceptible; I, intermediate; R, resistant.

| **Antibiotics** | **MIC (µg ml⁻¹)** | | | |
| --- | --- | --- | --- | --- |
|  | **1** | **2** | **3** | **4** |
| Penicillin | 0.19 (S) | 0.5 (S) | 1 (I) | 0.38 (S) |
| Ampicillin | 0.25 (S) | 0.25 (S) | 0.5 (S) | 0.5 (S) |
| Amoxicillin-clavulanate | 0.5/0.25 (S) | 0.5/0.25 (S) | 0.75/0.375 (S) | 0.75/0.375 (S) |
| Piperacillin-tazobactam | 1.5/4 (S) | 3/4 (S) | 4/4 (S) | 3/4 (S) |
| Clindamycin | **8 (R)** | **8 (R)** | **256(R)** | **48 (R)** |
| Ertapenem | 0.94 (S) | 0.25 (S) | 0.25 (S) | 0.25 (S) |
| Imipenem | 0.75 (S) | 1.5 (S) | 0.5 (S) | 1.5 (S) |
| Metronidazole | 0.19 (S) | 0.094 (S) | 0.64 (S) | 0.19 (S) |
